# Charge Engineering of Mo_2_C@Defect-Rich N-Doped Carbon Nanosheets for Efficient Electrocatalytic H_2_ Evolution

**DOI:** 10.1007/s40820-019-0279-8

**Published:** 2019-06-01

**Authors:** Chunsheng Lei, Wen Zhou, Qingguo Feng, Yongpeng Lei, Yi Zhang, Yin Chen, Jiaqian Qin

**Affiliations:** 1 State Key Laboratory of Powder Metallurgy, Central South University, Changsha, 410083 People’s Republic of China; 2 College of Environmental and Safety Engineering, Changzhou University, Changzhou, 213164 People’s Republic of China; 3 Key Laboratory of Advanced Technologies of Materials, Ministry of Education, and Institute of Materials Dynamics, Southwest Jiaotong University, Chengdu, 610031 Sichuan People’s Republic of China; 4 Hunan Provincial Key Laboratory of Chemical Power Sources, College of Chemistry and Chemical Engineering, Central South University, Changsha, 410083 People’s Republic of China; 5 Metallurgy and Materials Science Research Institute, Chulalongkorn University, Bangkok, 10330 Thailand

**Keywords:** Molybdenum carbide, Nitrogen-doped carbon nanosheets, Charge engineering, Graphene wave, Hydrogen evolution reaction

## Abstract

Charge engineering of carbon materials with many defects shows great potential in electrocatalysis, and molybdenum carbide (Mo_2_C) is one of the noble-metal-free electrocatalysts with the most potential. Herein, we study the Mo_2_C on pyridinic nitrogen-doped defective carbon sheets (MoNCs) as catalysts for the hydrogen evolution reaction. Theoretical calculations imply that the introduction of Mo_2_C produces a graphene wave structure, which in some senses behaves like N doping to form localized charges. Being an active electrocatalyst, MoNCs demonstrate a Tafel slope as low as 60.6 mV dec^−1^ and high durability of up to 10 h in acidic media. Besides charge engineering, plentiful defects and hierarchical morphology also contribute to good performance. This work underlines the importance of charge engineering to boost catalytic performance.

**Electronic supplementary material:**

The online version of this article (10.1007/s40820-019-0279-8) contains supplementary material, which is available to authorized users.

## Highlights

- The Mo_2_C modified carbon nanosheets produce a graphene wave structure to form localized charges and further enhance the N-doping effect.
- The optimal sample shows a Tafel slope as low as 60.6 mV dec^−1^ and high durability up to 10 h in acidic media.

**Electronic supplementary material:**

The online version of this article (10.1007/s40820-019-0279-8) contains supplementary material, which is available to authorized users.

## Graphical abstract

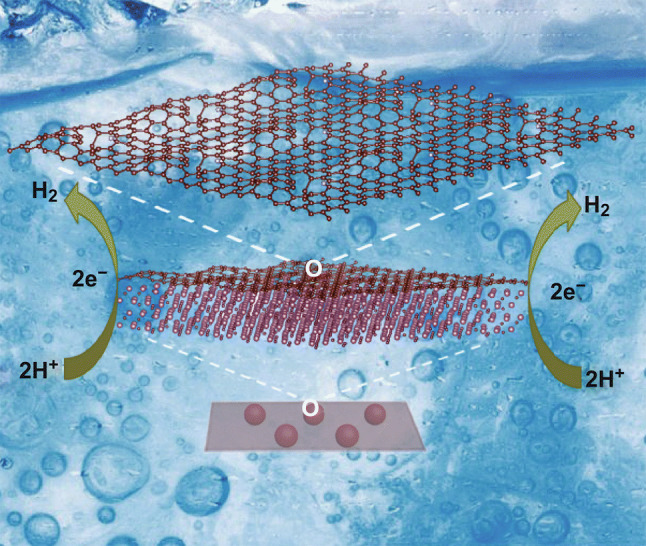

## Introduction

With the intensification of global energy consumption and severe environmental deterioration, sustainable and environmentally friendly approaches have aroused increasing interest [1]. Among ongoing attempts to produce clean fuels, the electrolysis of water to produce H_2_ is attractive [2–6]. The key to this problem is to seek an effective electrocatalyst to minimize the overpotential for hydrogen evolution reaction (HER). To replace Pt-based noble metals, abundant earth catalysts have received great attention [7–11]. Because of their similar electronic structure and catalytic behaviors to Pt [12], Mo-based compounds [13, 14], especially molybdenum carbide [15], have drawn tremendous fascination. On the other hand, the electronic structure significantly affects the interaction between the catalyst surface and reactants [4, 16–20]. Charge engineering is an important strategy to regulate the surface/interface behaviors involved in catalysis. For example, Sasaki et al. [21] found that carbide ligand changed the d-electron configuration of Mo_2_C to moderate Mo–H binding energy, leading to enhanced-HER activity.

Recently, researchers have paid great attention to nitrogen-doped defective carbon materials for electrocatalysis [22–26]. The defects not only play an important role in the adsorption/desorption during reaction, but also change the electrical conductivity of the catalysts to regulate the electronic structure [27]. However, related work on the application of MO_2_C to pyridinic N-doped carbon is limited. Furthermore, for gas-involving electrocatalysis, the hierarchical morphology is also important to optimize the gas/mass transport [16].

Considering the above-mentioned observations, Mo_2_C@ defect-rich N-doped carbon nanosheets (MoNCs) were developed. The theoretical results imply that the introduction of Mo_2_C produces a graphene wave structure, which to a degree behaves like N doping to form localized charges. As expected, the catalyst shows high-electrocatalytic HER activity with a Tafel slope as low as 60.6 mV dec^−1^ and stability up to 10 h in acidic media, making it one of the best Mo_2_C electrocatalysts. The multifold design, including charge engineering and nanoarchitecture construction, contributes to the HER performance.

## Experimental Section

### Materials

Ammonium heptamolybdate ((NH_4_)_6_Mo_7_O_24_·4H_2_O), melamine (C_3_H_6_N_6_), sucrose, and sulfuric acid (H_2_SO_4_, 98%) were bought from Tianjin Kaida Chemical Factory, Tianjin Kermel Chemical Factory, Sinopharm Chemical Reagent Co., Ltd., and Beijing Chemical Factory, respectively. Nafion solution (5 wt.%, Dupont D520) and Pt/C (20 wt.%, JM) were bought from Shanghai Hesen Electric Co., Ltd.

### Synthesis

Graphitic carbon nitride (g-C_3_N_4_) was prepared by simple calcination. A certain quantity of melamine was placed in an alumina crucible (100 mL) with a cover and then heated at 550 °C for 4 h in a muffle furnace (2.3 °C min^−1^). Subsequently, the obtained light-yellow solid product was milled into a powder state and sealed for later use. Next, ammonium heptamolybdate, sucrose, and g-C_3_N_4_ were mixed together (1:2:2, mass ratio). The homogeneous mixture was placed in an alumina crucible and then transferred to the center of the tube furnace. After pumping and purging the system three times with N_2_ flow, it was heated to 800 °C (3 °C min^−1^) and maintained at 800 °C for 6 h under flowing N_2_. The obtained black sample, called MoNCs (Mo_2_C@N-doped carbon sheets; the mass ratio of g-C_3_N_4_ and ammonium heptamolybdate is 2), was then ground to a fine powder without further treatment. For comparison, MoNCs-0, MoNCs-1, and MoNCs-5 (the mass ratio of g-C_3_N_4_ and ammonium heptamolybdate is 0, 1, 5), as counterparts, were also prepared in the same way.

### Materials Characterization

X-ray diffraction (XRD) was applied on Siemens D-5005 with Cu Kα radiation (2*θ* = 0.02° per step). X-ray photoelectron spectra (XPS) were performed with an Al Kα source (Thermo Scientific ESCALAB Ka+). The transmission electron microscope (TEM) operated on JEM-2100 at 200 kV. The nitrogen adsorption isotherm (ASAP 2020 at 77 K, USA) was recorded by the Brunauer–Emmett–Teller (BET) equation and Barrett–Joyner–Halenda (BJH) model. The Raman spectrum was measured on LabRAM HR800. Thermogravimetric analysis (TGA) was carried out on an SDT Q600 (TA) instrument under air flow (10 °C min^−1^) up to 700 °C. The product transformed according to the following reaction: Mo_2_C + 4O_2_ = 2MoO_3_ + CO_2_ [28].

### Computational Details

In this work, nanosheets with Mo_2_C nanoparticles have been modeled as single-layer graphene in a 6 × 6 × 1 supercell, and Mo_2_C in a 5 × 5 × 2 supercell. The cell and atomic coordinates are fully relaxed based on the density functional theory implemented in the Vienna Ab-initio Simulation Package (VASP) v5.3.5 [29–31] with grimme-D2 correction [32], where the PBE functional [33] and PAW pseudopotential [34, 35] have been used. The criteria of convergence of energy and force have been taken as 1 × 10^−6^ eV and 0.01 eV Å^−1^, respectively, the energy cutoff is set to 450 eV, and a 3 × 3×1 k-mesh is used to sample the Brillouin zone [36, 37]. The parameters are comparable to those in Refs. [36, 37]. In the core-level shift (CLS) calculation, the final state approximation has been adopted.

### Electrochemical Measurements

The HER measurements were performed in a typical three-electrode cell in 0.5 M H_2_SO_4_ using a CHI 660e electrochemical station (Shanghai Chenhua Co., China) at room temperature. A glassy carbon electrode (GCE) 8 mm in diameter, a saturated calomel electrode (SCE), and a graphite rod electrode were used as the working electrode, reference electrode, and counter electrode, respectively. All of the potentials were converted to the potential *versus* the reversible hydrogen electrode (RHE) according to *E* (RHE) = *E* (SCE) + 0.241 + 0.059 pH. The working electrode was fabricated as follows: A catalyst ink was prepared by dispersing 6 mg of catalyst into a mixed solution including 40 μL Nafion solution and 1 mL of 3:1 v/v water/isopropanol via sonication for at least 1 h to form a homogeneous ink. Then, 20 μL of well-dispersed catalyst ink was drop-casted on the glassy carbon electrode, producing a ~ 0.23 mg cm^−2^ loading for all samples, and the modified GC electrode was then dried at 50 °C in a drying oven for the following test. Before data collection, all working electrodes were pretreated by cyclic voltammetric scanning in 0.5 M H_2_SO_4_ solution to activate the electrodes. The electrochemical impedance spectroscopy (EIS) measurements were tested in 0.5 M H_2_SO_4_ solution with open-circuit voltage at a frequency from 10 mHz to 100 kHz at an amplitude of 5 mV.

## Results and Discussion

**Fig. 1:**
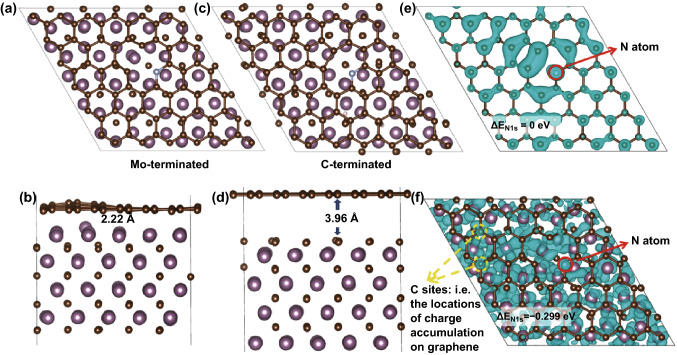
The **a** top and **b** side views of Mo-terminated Mo_2_C@N-doped graphene with pyridinic N dopant; the **c** top and **d** side views of C-terminated Mo_2_C@N-doped graphene with pyridinic N dopant; the charge distribution of **e** the unpaired electron of pyridinic N-doped graphene; and **f** Mo-terminated Mo_2_C@N-doped graphene with pyridinic N dopant. The N atom is circled in red. The locations of charge accumulation on graphene are circled in yellow. The figures are plotted with VESTA [39]

We combined Mo_2_C and N-doped graphene and theoretically simulated its atomic structure with first-principles calculation. Due to the limitation of computations, we used a slab of Mo_2_C covered with one layer of graphene to simulate part of the interface region of Mo_2_C and graphene. The vacuum was added in the direction perpendicular to the graphene plane, and periodicity was kept in the other two directions. According to the literature [37, 38], the β-Mo_2_C is a meta-stable structure at high temperature, where the Mo atoms are packed in a hexagonal close-packed structure, and the C atoms randomly occupy half of the octahedral interstitial sites (Fig. 1). As noted, the Mo_2_C@N-doped graphene has two types of structures: The Mo-terminated surface adhered to the graphene, and the C-terminated surface adhered to the graphene. We found that in Mo-terminated Mo_2_C@N-doped graphene, the graphene was approximately 2.22 Å above the Mo_2_C (Fig. 1b). Interestingly, the phenomenon of a graphene wave was observed, which should be attributed to the mismatch of the graphene and Mo_2_C unit cell. We have reason to believe that the graphene wave could introduce more localized charge density on some C sites, as indicated by the yellow circles (Fig. 1f), leading to the redistribution of electrons on the graphene and formation of a gradient of charge density to increase active sites. For C-terminated Mo_2_C@N-doped graphene, the graphene is found to be self-reorganized in the bulk defect region and approximately 3.96 Å above Mo_2_C (Fig. 1d).

Here, a Mo_2_C slab covered with one layer of graphene was used to simulate the real case, in which Mo_2_C nanoparticles are enclosed in nanosheets. Given that the nanoparticles have various shapes and sizes, the structural change and charge localization on the graphene should be more significant, as the nanoparticles have curved and highly indexed surfaces. On the other hand, with the increase in the number of nanosheets, the effect weakens. However, for a nanoparticle with two or three layers of nanosheets, the predicted phenomena should occur and contribute to the catalysis. Moreover, to clarify the influence of Mo_2_C on N-doped carbon sheets (NCS) further, we computed the N1*s* CLS within pyridinic N-doped graphene and Mo_2_C@N-doped graphene. For Mo-terminated Mo_2_C@N-doped graphene, the CLS moves forward to a higher energy (Figs. 1e, f and S1). The N1*s* core level deepens, which means that N achieved more electrons localized on the N atom. This will strengthen the N-doping effect and further promote the HER activity. Apart from these, we also calculated three more types of N doping in the Mo_2_C region within Mo_2_C@N-doped graphene, as shown in Fig. S1b–d. All three types of structures yield rather smaller binding energies for N1*s* core electrons.

**Fig. 2:**
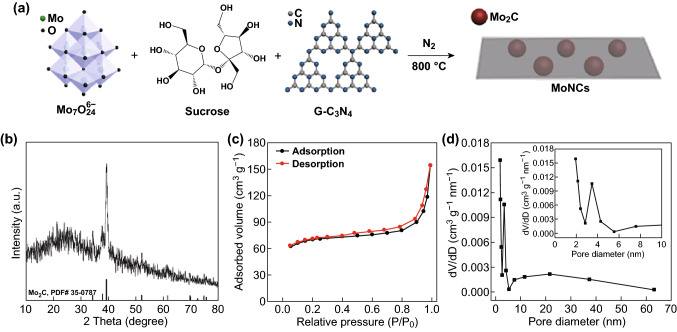
**a** Illustration of the synthesis route, **b** XRD pattern, **c** nitrogen adsorption–desorption isotherms, and **d** the corresponding pore size distribution curves of MoNCs

The synthesis route of MoNCs is shown in Fig. 2a. The carbonization of sucrose and the decomposition of ammonium heptamolybdate and g-C_3_N_4_ were integrated, rendering the simultaneous formation of uniformly dispersed Mo_2_C and N-doped carbon nanosheets. The synthesis avoids sophisticated or hazardous processes and expensive precursors. Figure 2b clearly displays the diffraction peaks at 34.4°, 38.0°, and 39.4°, attributed to the (100), (002), and (101) plane of hexagonal β-Mo_2_C (PDF# 35-0787), respectively [40]. No additional peaks were observed except for the (002) diffraction peak of graphite at ~ 26° [41]. Then, the N_2_ adsorption–desorption measurement (Fig. 2c) was carried out to investigate the specific surface area and pore structure. The typical type-IV isotherm curve is noted according to the Brunauer–Deming–Deming–Teller classification [42], verifying the presence of mesopores [43]. The BET surface area (*S*_BET_) is evaluated as 216.4 m^2^ g^−1^. The pore size distribution curve indicates the presence of micropores and mesopores, which are probably caused by the released molecules (H_2_O, CO_2_, C_2_N_2_^+^, C_3_N_2_^+^, C_3_N_3_^+^, etc.) during the carbonization of sucrose [44, 45] and the decomposition of g-C_3_N_4_ [46]. There is no doubt that the hierarchically porous structure will supply adequate diffusion passageways to strengthen the mass transfer [47].

**Fig. 3:**
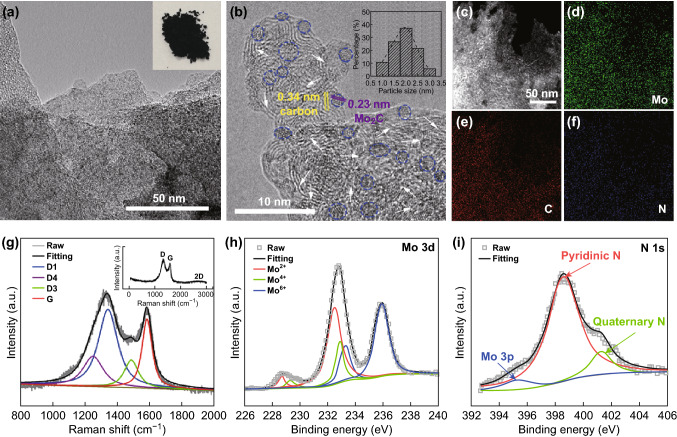
**a** TEM and **b** HRTEM images of MoNCs. Inset in **a** shows the optical photograph of Mo_2_C; inset in **b** shows the size distribution of Mo_2_C. **c** High-angle annular dark-field scanning transmission electron microscopy (HAADF-STEM) and **d–f** corresponding elemental mapping images. **g** Curve-fitting result of the Raman spectrum and the **h** Mo 3d and **i** N 1 s XPS spectra of MoNCs

In Fig. 3a, b, the typical TEM and HRTEM images reveal that MoNCs mainly consisted of a large amount of 1–3-nm Mo_2_C (inset in Fig. 3b) particles wrapped in thin carbon nanosheets. The lattice spacing is ~ 0.23 nm, corresponding to the distance between the (101) crystal planes of β-Mo_2_C. The thin carbon nanosheets (3–5 graphene layers) with lattice spacing of ~ 0.34 nm not only inhibit the agglomeration of Mo_2_C, but also guarantee the fast electron transfer and effective exposure of active phases. Massive defects were also noted (white arrows). The elemental mapping (Fig. 3c–f) result indicates that the Mo, N, and C atoms were homogeneously distributed. The Raman spectrum (Fig. 3g) also displays the typical D-band and G-band at 1341 and 1583 cm^−1^, respectively. The D1, D3, D4, and G peaks were fitted [48]. The high value (2.16) of *I*_D1_/*I*_G_ implies abundant defects, which are believed to enhance the electrocatalytic activity [49]. The Mo_2_C content in MoNCs determined by TGA is ~ 50.0 wt.% (Fig. S2). X-ray photoelectron spectroscopy (XPS) was used to further characterize the composition and chemical state of each element. As seen in Fig. S3, the survey XPS spectrum of the MoNCs shows obvious signals of elemental Mo, C, and N, which is consistent with the elemental mapping result above. The Mo 3d XPS spectrum (Fig. 3h) was deconvoluted into six peaks, corresponding to Mo^2+^ (228.6 and 232.4 eV), Mo^4+^ (229.3 and 232.8 eV), and Mo^6+^ (233.2 and 235.9 eV) species [50]. Mo^2+^ comes from Mo_2_C, which serves as the active sites for HER [51, 52]. In Fig. S4, the main peak at 284.6 eV in the deconvoluted C1*s* spectrum implies that graphite carbon is the majority species [53]. The N1*s* XPS spectrum was deconvoluted into two peaks at 401.3 and 398.6 eV (Fig. 3i), corresponding to the quaternary N (20%) and pyridinic N (80%), respectively. The high pyridinic N content will be favorable for HER [54]. Moreover, the element contents in the MoNCs were calculated and summarized in Table S2.

**Fig. 4:**
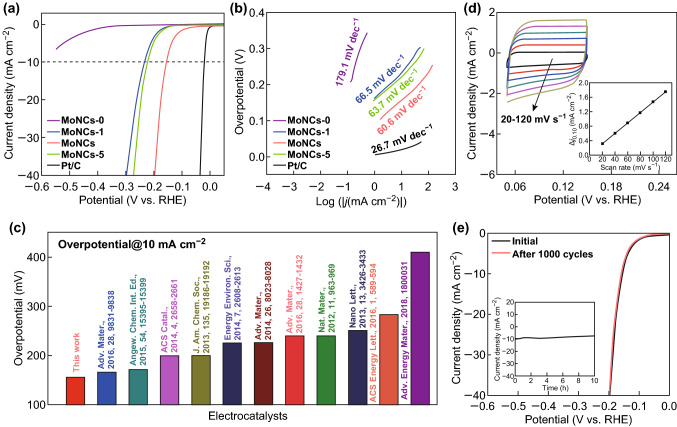
**a** LSV curves of a series of samples measured in 0.5 M H_2_SO_4_ solution. Scan rate: 2 mV s^−1^. **b** The corresponding Tafel plots. **c** The overpotentials delivered at 10 mA cm^−2^ of Mo-based electrocatalysts in recent years. **d** CV curves of MoNCs. Inset shows the capacitive current measured at 0.10 V versus RHE. **e** Stability test for MoNCs by CV scanning; inset shows the time-dependent current density curve at a static overpotential of 157 mV for 10 h

The electrocatalytic HER performance was investigated. For comparison, control samples synthesized with different mass ratios of starting materials were also tested. In Fig. 4a, the MoNCs show a low onset overpotential of 83 mV and the lowest overpotential of 157 mV @ 10 mA cm^−2^, exhibiting the best HER activity among the four control samples. In addition, the influence of loading mass on the HER activity was also studied (Fig. S5). The linear sweep voltammetry (LSV) curves of the MoNCs are also provided when the loading mass increased from 0.115 to 0.460 mg cm^−2^, indicating the enhanced-HER performance with more loadings. To demonstrate the HER mechanism, the linear sections of the Tafel plots were fitted to the Tafel equation (*η* = *a* + *b* log (*j*), where *a* is the intercept, *b* is the Tafel slope, and *j* is the current density), as shown in Fig. 4b. The MoNCs achieve the smallest Tafel slope of 60.6 mV dec^−1^ among the four samples (Table S3), suggesting that HER can likely proceed through the Volmer–Heyrovsky mechanism [55]. Remarkably, the exchange current density (*j*_0_) for MoNCs, calculated by extrapolating the Tafel plot to an overpotential of 0 mV (Fig. S6), was also the highest (2.65 × 10^−2^ mA cm^−2^). Compared to the previously reported Mo_2_C-based non-precious-metal catalysts, the excellent HER performance makes our sample one of the most promising electrocatalysts (Fig. 4c, Table S3). MoNCs show almost no current loss after 1000 CV cycles (Fig. 4e). Furthermore, the current density exhibits a negligible degeneration at a static overpotential of 157 mV after 10 h of constant operation, verifying the high durability.

**Fig. 5:**
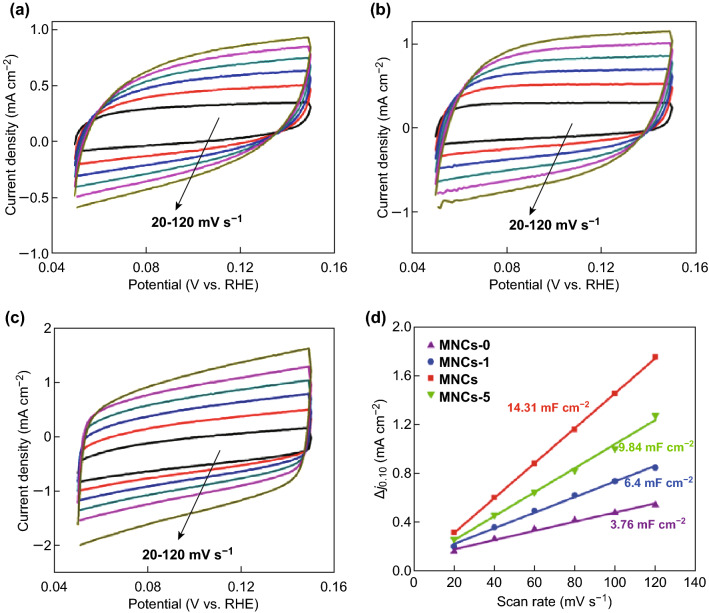
CV curves of **a** MoNCs-0, **b** MoNCs-1, and **c** MoNCs-5. **d** The capacitive current of those four catalysts measured at 0.10 V versus RHE is plotted as a function of scan rate

The effective electrochemical surface area (ECSA) was evaluated by the electrochemical double-layer capacitance (EDLC, *C*_dl_) (Figs. 4d, 5). The *C*_dl_ measured at 0.10 V for MoNCs was the highest (14.31 mF cm^−2^), indicating more active sites. The EIS was also tested (Fig. S7). The Nyquist plot of the MoNCs displays a small semicircle among them, suggesting lower impedance to accelerate the charge transfer during HER. Furthermore, the turnover frequency (TOF) of MoNCs was estimated [56] (see more details from supporting information). In Fig. S8, the MoNCs achieved a TOF of 0.07 and 1.12 s^−1^ at overpotential of 150 and 250 mV, respectively. The values were much higher than those of other catalysts, indicating more active sites.

## Conclusion

In summary, we illustrate charge engineering of Mo_2_C@ defect-rich N-doped carbon nanosheets for electrocatalytic H_2_ evolution. The calculation result indicates that the introduction of Mo_2_C induces a graphene wave structure, which behaves like N doping to form localized charges for the first time. The thin carbon nanosheets, combined with plentiful defects, facilitate the fast electron transfer and effective exposure of active phases. As a result, the sample displays a Tafel slope as low as 60.6 mV dec^−1^ and high durability up to 10 h in acidic media, featuring excellent HER catalytic activity and stability. Our work emphasizes the importance of charge engineering in electrocatalysis.

## Electronic supplementary material

Below is the link to the electronic supplementary material.

Supplementary material 1 (PDF 637 kb)
