# Supplementary material for: Charge Engineering of Mo2C@Defect-Rich N-Doped Carbon Nanosheets for Efficient Electrocatalytic H2 Evolution
Source: Nanomicro Lett. 2019 Jun 1;11:45. doi: 10.1007/s40820-019-0279-8 (PMC7770866; doi:10.1007/s40820-019-0279-8)
Supplement: Supplementary file 1 — Supplementary material 1 (PDF 637 kb) [file 40820_2019_279_MOESM1_ESM.pdf]

Supporting Information for

Charge Engineering on Mo_2C@defect-rich N-doped Carbon Nanosheets for Efficient ELectrocatalytic H_2 Evolution

Chunsheng Lei[1, 2], Wen Zhou[1, 2], Qingguo Feng[3], Yongpeng Lei[1, 4, *], Yi Zhang[4], Yin Chen[4], Jiaqian Qin[5]

[1]State Key Laboratory of Powder Metallurgy, Central South University, Changsha 410083, People's Republic of China

[2]College of Environmental & Safety Engineering, Changzhou University, Changzhou 213164, People's Republic of China

[3]Key Laboratory of Advanced Technologies of Materials, Ministry of Education, and Institute of Materials Dynamics, Southwest Jiaotong University, Chengdu, Sichuan 610031, People's Republic of China

[4]Hunan Provincial Key Laboratory of Chemical Power Sources, College of Chemistry and Chemical Engineering, Central South University, Changsha 410083, People's Republic of China

[5]Metallurgy and Materials Science Research Institute, Chulalongkorn University, Bangkok 10330, Thailand

Chunsheng Lei, Wen Zhou, and Qingguo Feng contributed equally to this work

*Corresponding author. E-mail: lypkd@163.com (Y. Lei)

S1 ECSA and TOF Calculation

The electrochemical active surface area (ECSA) can be estimated using the capacitance (C). The specific capacitance for a flat surface is generally found to be in the range of 20~60 μF cm^{-2}. In the following calculations of the ECSA and turnover frequency (TOF), 40 μF cm^{-2} was used as literatures.

Generally, we calculated the ECSA of each sample according to the capacitance measurement (Eq. (S1)):

$$\text{ECSA} = \frac{C}{40\ \mu\text{F cm}^{-2}\ \text{per cm}^{-2}} \quad \text{(S1)}$$

Where, C represents the capacitance; 40 μF cm^{-2} was used in the above formula. To further comprehend the inherent electrocatalytic performance of each sample, the turnover frequency (TOF) was also estimated by Eq. (S2):

$$\mathrm{TOF} = \frac{\text{number of total hydrogen turnover per cm}^{-2}}{\text{number of active sites per cm}^{-2}} = \frac{\#_{\mathrm{H_2}} \times |j|}{\text{active sites} \times \text{ECSA}} \qquad \text{(S2)}$$

The number of total hydrogen turnovers ($\#_{\mathrm{H_2}}$) was calculated from the current density according to Eq. (S3):

$$\#_{\mathrm{H_2}} = \left(j\,\frac{\mathrm{mA}}{\mathrm{cm^2}}\right)\left(\frac{1\mathrm{C\,s^{-1}}}{1000\ \mathrm{mA}}\right)\left(\frac{1\mathrm{mol\ H_2}}{96485.3\ \mathrm{C}}\right)\left(\frac{1\mathrm{mol\ H_2}}{2\mathrm{mol\ of\ e^-}}\right)\left(\frac{6.02 * 10^{23}\mathrm{H_2\ moleculars}}{1\ \mathrm{mol\ H_2}}\right)$$

$$= 3.12 * 10^{15}\,\frac{\mathrm{H_2/s}}{\mathrm{cm^2}}\ \text{per}\ \frac{\mathrm{mA}}{\mathrm{cm^2}} \qquad \text{(S3)}$$

The number of active sites per surface area was calculated according to the crystal data as Eq. (S4):

$$\text{Active sites}_{\mathrm{Mo_2C}} = \left(\frac{2\ \text{atom/unit cell}}{37.2\ \text{Å}^3\text{/unit cell}}\right)^{\frac{2}{3}} = 1.42 * 10^{15}\ \text{atom per cm}^2 \qquad \text{(S4)}$$

S2 Supplementary Figures and Tables

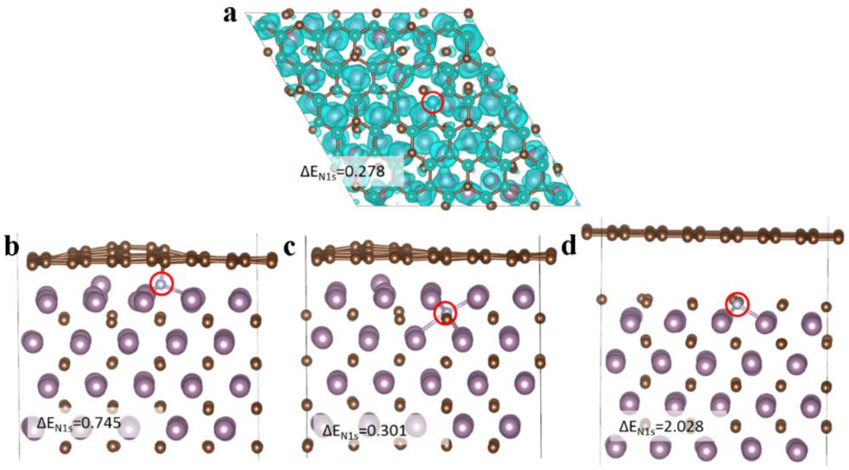

Fig. S1 The charge distribution of **a** C-terminated Mo_2C@N doped graphene with pyridinic-N dopant. Illustration of Mo_2C@N doped graphene with N **b** on surface and **c** in bulk Mo-terminated Mo_2C and **d** in top surface C layer of C-terminated Mo_2C. Red circled is N atom. The figures are plotted with VESTA

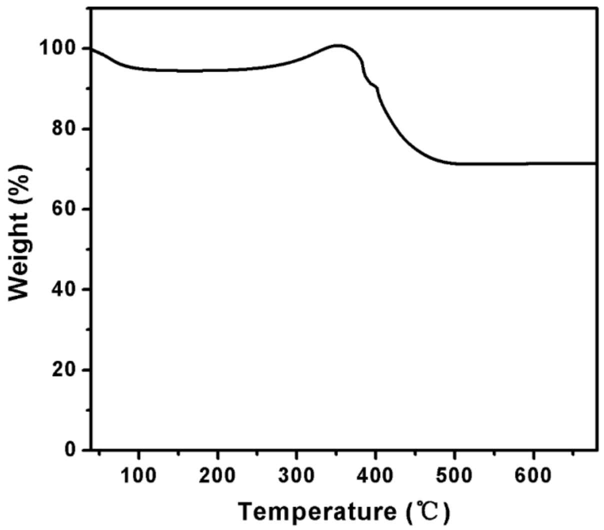

Fig. S2 TGA curve of MoNCs under air flow

As depicted in TGA curve, the initial weight losses below 100 °C is due to the water evaporation, followed by a gradual oxidation and transformation from Mo_2C to MoO_3. When heating to above 350 °C, the sample appeared markedly weight loss as a result of the combustion of carbon. As the temperature continuously after raised to 500 °C, the weight loss nearly showed no fluctuation, indicating the only remaining product was MoO_3 (about 70 wt% from the TGA curve). The Mo_2C content in MoNCs was calculated by Eq. (S5):

m (Mo_2C) = m (residual mass) × M (Mo_2C)/(2 × M (MoO_3))

= 70% × 204 / (2 × 144)

= 49.58 %

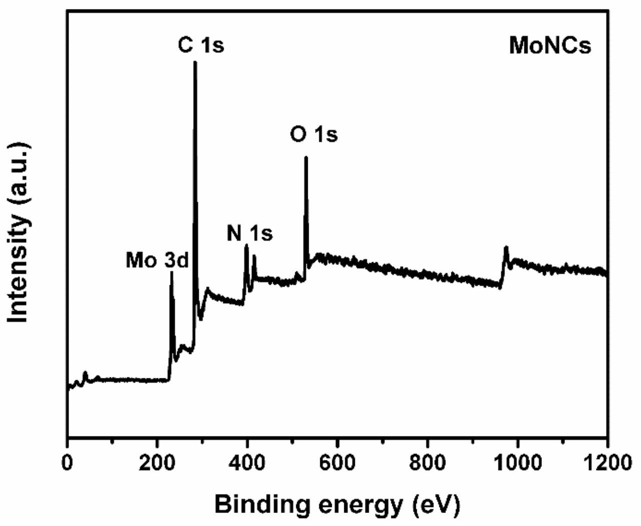

Fig. S3 The survey spectrum of MoNCs

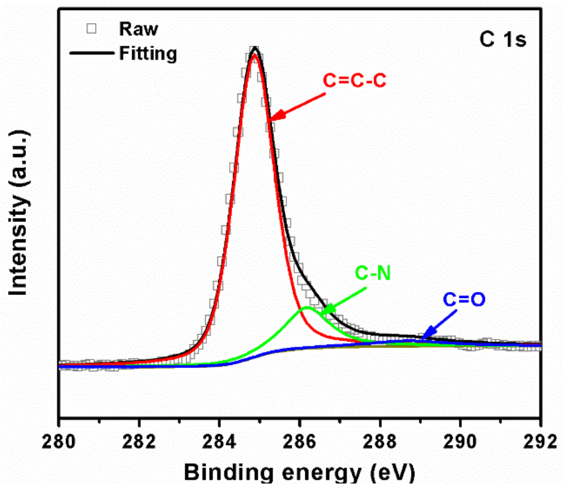

Fig. S4 C 1s XPS spectrum of MoNCs

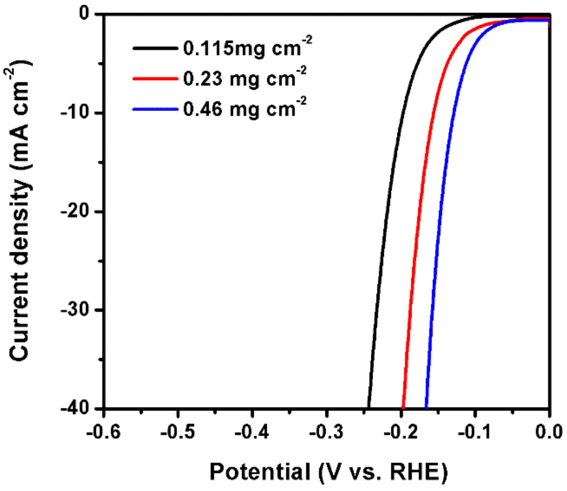

Fig. S5 LSV curves of MoNCs electrocatalyst with different loading amounts

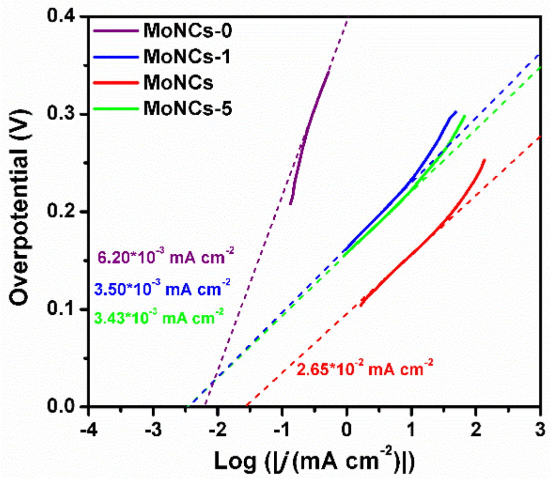

Fig. S6 The calculated exchange current density by Tafel plot

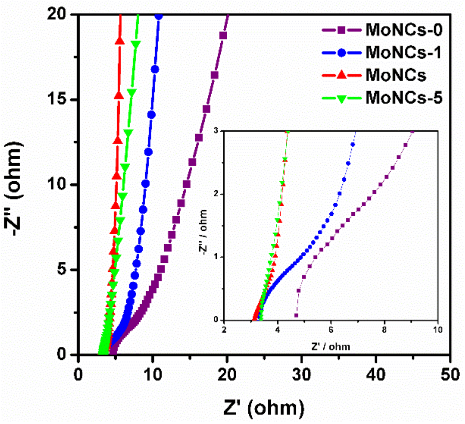

Fig. S7 Nyquist plots of electrochemical impedance spectroscopy for different samples

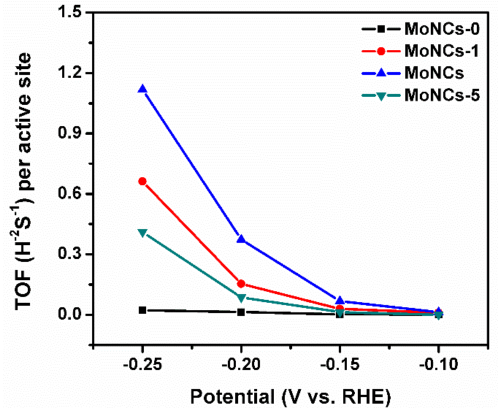

Fig. S8 TOF values of different samples

Table S1 The atom percentage of different elements in $MoNC_{s-x}$ by XPS measurement

Samples	Mo	C	N	O
MoNCs-0	4.45	79.17	0	16.38
MoNCs-1	4.02	78.26	1.34	16.34
MoNCs	3.4	81.82	1.46	13.34
MoNCs-5	0.9	91.5	1.88	5.72

Table S2 Electrochemical parameters of different comparison samples

Samples	Onset potential (mV)	η_{10} (mV)
MoNCs-0	391	-
MoNCs-1	163	233
MoNCs	83	157
MoNCs-5	158	223

Table S3 The comparison of HER performances in 0.5 M H_2SO_4 and synthesis method with other Mo-based catalysts

**Catalysts**	**Onset potential (mV)**	η_{10} **(mV)**	**Tafel slope (mV dec^{-1})**	j_0 **(mA cm^{-2})**	**Synthesis method**	**Refs.**
MoNCs	83	157	60.6	2.65×10^{-2}	a one-step pyrolysis	This work
MoP	~120	240	66	-	annealed MoS_2 and red phosphorus in Ar/H_2	*Adv. Mater.*, **2016**, 28, 1427-1432
$MoSe_2$/Mo core-shell nanoscrews	89	166	34.7	-	two-stage sputtering processes for preparing Mo film, then low-temperature plasma-assisted selenization process for producing sample	*Adv. Mater.*, **2016**, 28, 9831-9838
Monolayer MoS_2/3D gold	118	226	46	-	chemically dealloying prepare NPG, then prepare MoS_2 by CVD method	*Adv. Mater.*, **2014**, 26, 8023-8028
$Co_{0.6}Mo_{1.4}N_2$	-	200	-	0.23	multiple step	*J. Am. Chem. Soc.*, **2013**, 135, 19186-19192
Double-gyroid MoS_2	150-200	240	50	$1.3\text{-}6.9\times10^{-4}$	TMDC based on Mo, Se, and Te with different composition were grown by MBE on HOPG under UHV conditions	*Nat. Mater.*, **2012**, 11, 963-969
Mo_2C nanotubes	-	172	62	1.7×10^{-2}	by carburizing Mo-polydopamine nanotubes under a N_2 gas flow	*Angew. Chem. Int. Ed.*, **2015**, 54, 15395-15399
$MoSe_2$/carbon fiber paper	110	250	59.8	3.8×10^{-4}	electrodeposition of Mo into a silica template followed by sulphidization with H_2S	*Nano Lett.*, **2013**, 13, 3426-3433
$MoSe_{0.12}Te_{1.79}$	180	410	62	-	via a solid-state reaction under Ar using $(NH_4)_6Mo_7O_{24}\cdot4H_2O$ and ALG	*Adv. Energy Mater.*, **2018**, 1800031
Mo_2C/GCSs	120	200	62.6	1.25×10^{-2}	Mo_2CTx and Ti_2CTx MXenes were synthesized by HF etching of their parent ternary carbides, Mo_2Ga_2C and Ti_2AlC, by removing the Ga and Al atoms, respectively	*ACS Catal.*, **2014**, 4, 2658-2661
Mo_2CT_x MXenes	~100	283	70	-	chemical vapor deposition, including by using a microwave-assisted intercalation method.	*ACS Energy Lett.*, **2016**, 1, 589-594
NiMoNx/C (nitrides)	157	225	35.9	-	Mo reacted with sulfur or selenium vapor	*Energy Environ. Sci.*, **2014**, 7, 2608-2613
